# Multiple antibiotic resistances among Shiga toxin producing *Escherichia coli* O157 in feces of dairy cattle farms in Eastern Cape of South Africa

**DOI:** 10.1186/s12866-015-0553-y

**Published:** 2015-10-16

**Authors:** Benson C. Iweriebor, Chinwe J. Iwu, Larry C. Obi, Uchechukwu U. Nwodo, Anthony I. Okoh

**Affiliations:** SA-MRC Microbial Water Quality Monitoring Centre, University of Fort Hare, Alice, 5700 South Africa; Applied and Environmental Microbiology Research Group, Department of Biochemistry and Microbiology, University of Fort Hare, Alice, 5700 South Africa; Academic and Research Division, University of Fort Hare, Alice, Eastern Cape South Africa

**Keywords:** *E.coli*, Shiga toxins, Antibiotic susceptibility, Cattle, STEC

## Abstract

**Background:**

Shiga toxin–producing *Escherichia coli* (STEC) O157:H7 is a well-recognized cause of bloody diarrhea and hemolytic-uremic syndrome (HUS). The ability of STEC strains to cause human disease is due to the production of Shiga toxins. The objectives of this study were to determinate the prevalence, serotypes, antibiotic susceptibility patterns and the genetic capability for Shiga toxin production in *Escherichia coli* (STEC) strains isolated from dairy cattle farms in two rural communities in the Eastern Cape Province of South Africa.

**Methods:**

Fecal samples were collected between March and May 2014, from individual cattle (*n* = 400) in two commercial dairy farms having 800 and 120 cattle each.

Three hundred presumptive isolates obtained were subjected to polymerase chain reactions (PCR) for identification of O157 serogroup and Shiga toxin producing genes (*stx*1*, stx*2) on genomic DNA extracted by boiling method. Susceptibility of the isolates to 17 antibiotics was carried out *in vitro* by the standardized agar disc-diffusion method.

**Results:**

Based on direct PCR detection, 95 (31.7 %) isolates were identified as O157 serogroup. The genetic repertoire for Shiga toxin production was present in 84 (88.42 %) isolates distributed as *stx*1 (37), *stx*2 (38) and *stx*1/2 (9) respectively while 11 of the isolates did not harbor Shiga toxin producing genes. Multiple antibiotic resistances were observed among the isolates and genetic profiling of resistance genes identified *bla*_ampC_ 90 %, *bla*_CMY_ 70 %, *bla*_CTX-M_ 65 %, *bla*_TEM_ 27 % and *tet*A 70 % and *str*A 80 % genes among the antimicrobial resistance determinants examined.

**Conclusion:**

We conclude that dairy cattle farms in the Eastern Cape Province are potential reservoirs of antibiotic resistance determinants in the province.

## Background

*Escherichia coli* is an important pathogen in cattle, medicine and public health [1], and Shiga toxin-producing strain (STEC) have emerged as important food-borne pathogens, especially serotype O157:H7. Human diseases caused by this serotype that produces STEC ranges from mild diarrhoea to haemorrhagic colitis and haemolytic uraemic syndrome (HUS) and typically it affects children, the elderly, and immunocompromised patients [2]. Healthy domestic ruminants such as cattle, sheep, and goats can harbor STEC and *E. coli* O157:H7 in their faeces and are thus natural reservoirs of these pathogens [3–5]. The pathogenicity of STEC resides in a number of virulence factors, including Shiga toxins (*Stx*1 and *Stx*2), intimin, enterohaemolysin, and the STEC autoagglutinating adhesin (*Saa*) [6]. Shiga toxin–producing (STEC) and enteropathogenic *Escherichia coli* (EPEC) represent two of the six different categories of diarrheagenic *E. coli* that can cause disease in humans [1]. STEC, which is defined by the production of two Shiga toxins, *Stx*1 and/or *Stx*2, is a zoonotic pathogen that is a major cause of diarrhea worldwide. *Stx*2 is more closely related to these diseases than *stx*1 [7]. There are three Stx1 subtypes (Stx1a, Stx1c, and Stx1d) and seven Stx2 subtypes (Stx2a, Stx2b, Stx2c, Stx2d, Stx2e, Stx2f, and Stx2g) according to the subtyping nomenclature proposal put forth at the 7th International Symposium on Shiga Toxin (Verocytotoxin)–Producing *Escherichia coli* Infection, held in Buenos Aires, in 2009.

The use of antimicrobial in animal feeds as growth promoters is common worldwide. Across the globe, a variety of antimicrobial agents are available for therapeutic use or as growth promotion in animals. Many studies have supported the claim that with the increased use of antimicrobial agents in animals and humans, an increased prevalence of resistant strains may be selected as a direct consequence of the antimicrobial use [8, 9]. Humans, via the food chain, ingest a lot of bacteria originating from food-producing animals, which have been recognized as major reservoirs of *E. coli* habouring CTX-M β-lactamase an enzyme that confers resistance to β-lactam antibiotics [23]. Sasaki [10] have reported a high prevalence of CTX-M β-lactamase encoding gene among *Enterobacteriaceae* in stool specimens from healthy asymptomatic volunteers in a rural community in Thailand. Concerns exist about the potential spread of the β-lactam-CTX-M genes from food animal products to humans through the food chain. CTX-M β-lactamase genes have been reported in *E. coli* from various food-producing animals worldwide raising a potential threat to public health [11–16]. Giving the frequent occurrence of O157 STEC as foodborne pathogen in North America and some parts of the western world, there is a clear need to gather data on the prevalence and distribution of STEC producing *E.coli* and the antibiotic resistance profiles of isolates of this organism recovered from faecal samples from commercial dairy cattle farms in the Eastern Cape of South Africa where pastoral farming is a major source of income for many families. This study therefore aimed at characterizing *E.coli* O157 isolates from fecal samples of two dairy cattle farms in the Eastern Cape Province of South Africa. Ability of isolates to produce Shiga toxin and their antibiotic susceptibility patterns as well as presence of some resistance determinants were screened by molecular approaches.

## Methods

### Ethical clearance

Ethical clearance was obtained from the University of Fort Hare ethics committee prior to sample collection and permission was sought from farmers from whose farms samples were collected.

#### Study population and sampling

Details on the study population and sampling procedures are as follows. Briefly, samples were collected from commercial dairy cattle farms in Nkonkobe District of Eastern Cape Province, South Africa. A total of 400 samples from two commercial dairy farms were collected for the study. Rectal fecal grab samples of approximately 10 g were collected from individual cattle using sterile gloves into appropriate capped containers. After collection, samples were shipped on ice to the University of Fort Hare Microbiology laboratory for immediate processing.

#### Preliminary sample processing

Approximately 1 g of each fecal sample was mixed in 9 ml of Trypticase soya broth (TSB) with 20 mg/L novobiocine and incubated for 6–8 h at 37 °C. This was streaked out onto sorbitol MacConkey agar (SMAC) supplemented with 1 mg/L potassium tellurite and incubated for 18–24 h at 37 °C. A pale colony each (sorbitol non- fermenters) was picked as presumptive *E. coli* O157 per sample. The pure colonies were each inoculated into separate TSB and incubated for 24 h at 37 °C from which glycerol stock was made and then stored at −80 °C for further analyses.

#### DNA extraction

Bacterial DNA was prepared as previously described by Bai [17]; briefly, bacterial culture from glycerol stock was resuscitated by an overnight growth in trypticase soya broth (TSB) (Oxoid Ltd, London, UK) at 37 °C with slight agitation. From this culture, 2 ml was centrifuged for 5 min at 14,000 rpm and the pellet was washed with normal saline (0.85 % NaCl). After the addition of 150 μl of rapid lysis buffer (100 mM NaCl, 10 mM Tris-HCl pH8.3, 1 mM EDTA pH9.0; 1 % Triton X-100), the suspension was votexed, boiled for 15 min, centrifuged at 10,000 rpm, supernatants collected in a DNase free Eppendorf tube and stored at −20 °C. These were then used as templates in all the polymerase chain reactions (PCRs) that were performed in this study.

#### Molecular serotyping and virulence typing

Molecular serotyping using the O-unit flippase gene (*wzx*) was performed: primers used for detection of O157 strains are shown in Table 1. *E. coli* O157:H7 ATCC 35150 served as the positive control. Specific primers were used to detect the presence of virulence genes encoding the Shiga-toxins (*stx*1 and *stx*2) as previously described by [17, 18]. Amplification was performed using 25 μl of PCR mix containing 5 μl of bacterial DNA purified as described above, 12 μl of 2X Dream Taq Master Mix (Thermo Scientific), 10pmol of both forward and reverse primers and 6 μl of water of PCR grade. The conditions for PCR amplification performed in a thermal cycler (BioRad) were 94 °C for 3 min, followed by 35 cycles of 93 °C for 60 s, either 55 °C for 60 s and 72 °C for 60 s. The final cycle was followed by an extension step at 72 °C for 7 min. The amplified products were visualized by standard gel electrophoresis using 5 μl of the PCR product on 2 % agarose gels in 0.5X TBE buffer (0.1 M Tris, 0.1 M boric acid and 0.002 M NaEDTA). Gels were stained using ethidium-bromide (1 mg/ml) and photographed under UV light in a transilluminator (ALLIANCE 4.7).

**Table 1:** Primers used in PCR detection of Shiga toxin genes and determination of serotypes

Target gene	Primer sequences 5′-3′	Size of product (bp)	Reference
*stx1*	STx1-F TTC GCT CTG CAA TAG GTA	555	[18]
	STx1-R TTC CCC AGT TCA ATG TAA GAT		
*stx2*	STx2-F GTG CCT GTT ACT GGG TTT TTC TTC	118	[18]
	STx2-R AGG GGT CGA TAT CTC TGT CC		
*rfbE*(O157)	rfbE-F TTT CAC ACT TAT TGG ATG GTC TCA A	88	[17]
	rfbE-R CGA TGA GTT TAT CTG CAA GGT GAT		

#### Antimicrobial drug susceptibility testing

We determined antimicrobial drug susceptibility by the disk-diffusion method on Mueller-Hinton agar plates as recommended by the Clinical Laboratory Standard Institute [19]. We tested the following antimicrobial agents: ampicillin 10 μg, tetracycline 30 μg, oxy-tetracycline 30 μg, which were used in the farms and the following agents used in the management of *E.coli* infections; amoxicillin/clavulanic acid 10 μg, cephalothin 30 μg, cefotaxime 30 μg, ceftazidime 30 μg, imipenem 10 μg, norfloxacin 10 μg, ciprofloxacin 5 ug, enrofloxacin 5 μg, amikacin 30 ug, chloramphenicol 10 μg, kanamycin 30 μg, streptomycin 10 μg, gentamicin 10 μg and sulfamethoxazole/trimethoprim (cotrimoxazole) 25 μg (Mast Diagnostics). Results obtained were used to classify isolates as being resistant or susceptible to a particular antibiotic using standard reference values [19].

#### PCR profiling of resistance genes

Template DNA was prepared as previously stated above. The β-lactamase genes *bla*_TEM_*, bla*_SHV,_*bla*_CMY-2_, *bla-*_CTX-M_, *bla-*_CTX-M1_, *bla-*_CTX-M9_ and *bla-*_ampC_ and two of the genes responsible for resistance to streptomycin (*str*A) and tetracycline (*tet*A) respectively were tested using specific primers in Table 2 as described previously in literatures (Forward et al., 2001, Guillaume et al., 2000, Thong et al., 2010). PCR was performed in a 25-μl mixture of 12 μl of 2X Dream Taq Master Mix (Thermo Scientific, Pittsburgh, PA, USA), 10 pmol of each forward and reverse primer and 6 μl water of PCR grade. The PCR mixture was subjected to a 3-min denaturation step at 94 °C, followed by 35 cycles of 45 s at 94 °C, 45 s at 55 °C/57 °C/50 °C depending on the primer set, and 60 s at 72 °C, and a final elongation step of 7 min at 72 °C. PCR products were separated by 120-V electrophoresis in a 2 % agarose gel containing ethidium bromide for 45 min, visualized in Alliance 4.7 transilluminator (ALLIANCE 4.7, Cambridge, United Kingdom) and photographed.

**Table 2:** Primers used to profile β-lactamases, *tet*(A) and *str*A genes in *Escherichia coli*

Gene detected	Primer sequence (5′- 3′)	Size (bp)	Reference
*blaTEM*	CF- TCG GGG AAA TGT GCG CG		
	DR-TGC TTA ATC AGT GAG GCA CC	971	[20]
*blaSHV*	OS-5 F- TTA TCT CCC TGT TAG CCA CC		
	OS-6R-GAT TTG CTG ATT TCG CTC GG	795	[21]
*blaCTX-M*	MA-1 F-SCS ATG TGC AGY ACC AGT AA	158	
	MA-2R- CCG CRA TAT GRT TGG TGG TG		[20]
*blaCTX-M* gp 9	M9UF-ATG GTG ACA AAG AGA GTG CA	863	
	M9LR-CCC TTC GGC GAT GAT TCT C		[20]
*blaCTX-M* gp 1	M13UF-GGT TAA AAA ATC ACT GCG TC	863	
	M13LR-TTG GTG ACG ATT TTA GCC GC		[20]
*blaampC*	AmpC1F-AAT GGG TTT TCT ACG GTC TG		
	AmpC2R-GGG CAG CAA ATG TGG AGC AA	191	[22]
*blaVEB*	casFF-CGA CTT CCA TTT CCC GAT GC		
	casBR -GGA CTC TGC AAC AAA TAC GC	1052	[23]
*blaCMY*	CF1F-ATGATGAAAAAATCGTTATGC		
	CF2R-TTGTAGCTTTTCAAGAATGCGC	507	[24]
tet(A)	TetA-F-GGCCTCAATTTCCTGACG		
	TetA-R-AAGCAGGATGTAGCCTGTGC	372	[25]
strA	strA-F-CTTGGTGATAACGGCAATTC		
	strA-R-CCAATCGCAGATAGAAGGC	548	[26]

## Results

### Serotyping

PCR-based molecular serotyping using primers designed for detection of O157 group identified 95 positive isolates as O157 serotype (gel not shown) out of the 320 presumptive isolates.

### PCR-based detection of virulence genes

The 95 molecularly confirmed *E.coli* O157 isolates were analyzed by PCR for their Shiga toxin producing capabilities. Table 3 shows PCR results of the different virulence genes for 84 (88.45 %) isolates detected among the 95 confirmed O157 isolates. The remaining 11 (11.55 %) though belonging to O157 serogroup, did not harbor Shiga toxin genes and were therefore regarded as non-STEC strains.

**Table 3:** Prevalence of Shiga toxin genes among the STEC O157 isolates

Serogroup	*stx*1	*stx*2	Prevalence of Shiga toxin genes
O157 (*N* = 84)	+	-	37 (44 %)
	-	+	38 (45.3 %)
	+	+	9 (10.7 %)

**Fig. 1:**
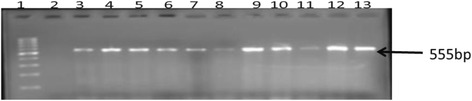
Gel image of amplified PCR products from study isolates with primers designed for *stx1* virulent gene. Lane 1 is the MWM (100 bp), lane 2 is the negative control (PCR mix without DNA) with lane 3 as positive control (*E*. coli ATCC 35150) while lanes 4 to 13 are *stx1* (555 bp) gene amplified from O157 isolates

Distribution of virulence genes among the 84 STEC strains showed that 37 (44 %) isolates possessed the *stx*1 gene as shown in Fig. 1, 38 (45.3 %) possessed the *stx*2 gene Figure not shown while 9 (10.7 %) had both *stx*1 and *stx*2.

### Antibiotic sensitivity patterns

Disk-diffusion susceptibility testing indicated high prevalence of multi-resistance to various antimicrobial agents among the 95 isolates: chloramphenicol 89.5 %, ampicillin 94.74 %, tetracyclin 96.84 %, oxytetracyclin 94.74 %, cefuroxime 82 %, ceftazidime 32 %, cephalothin 94.74 %, streptomycin 84.2 %, amikacin 6.3 %, kanamycin 5.3 %, amoxicillin/clavulanate 84.2 %, trimethoprim/sulfamethazole 84.2 %, norfloxacin 10.5 %, enrofloxacin 7.4 %, ciprofloxacin 12.6 %, and gentamycin 8.4 % while all the isolates were susceptible to imipenem as shown in Table 4 below. All isolates showed reduced susceptibility to several antimicrobials agents in the panel.

**Table 4:** Antibiotic susceptibility pattern of *E. coli* O157:H7 (*n* = 95)

Antibiotics class	Antimicrobial agents	Code	Potency (μg)	R	S
				N (%)	
Tetracycline	Tetracycline	T	30	92 (96.8)	3 (3.3)
	Oxytetracycline	OT	30	90 (94.7)	5 (5.3)
Penicillin	Ampicillin	AMP	10	90 (94.7)	5 (5.3)
	Amoxicillin/Claculanic acid	AUG	10	80 (84.2)	15 (15.8)
Cephalospirines	Cephalothin	KF	30	90 (94.7)	5 (5.3)
	Ceftazidime	CAZ	30	30 (32)	65 (78)
	Cefuroxime	CXM	30	78 (82)	17 (18)
Carbapenems	Imipenem	IMI	10	0 (0)	95 (100)
Phenicols	Chloramphenicol	CIP	10	85 (89.5)	10 (10.5)
Aminoglycosides	Amikacin	AK	30	6 (6.3)	89 (93.7)
	Kanamycin	K	10	5 (5.3)	90 (94.7)
	Streptomycin	S	10	80 (80.2)	15 (15.8)
	Gentamycin	GM	10	8 (8.4)	87 (91.6)
Quinolones	Ciprofloxacin	CIP	5	12 (12.6)	83 (87.4)
	Norfloxacin	NOR	10	10 (10.5)	85 (89.5)
	Enrofloxacin	ENR	10	7 (7.4)	88 (92.6)
Foliate pathway inhibitor	Sulfamethazole/Trimethoprim	TS	25	80 (80.2)	15 (15.8)

*R* Resistant, *S* Susceptible, *T* Tetracycline, *OT* Oxytetracycline, *AMP* Ampicillin, *AUG* Amoxilcillin/clavulanic acid, *KF* Cephalothin, *CXM* Cefuroxime, *CAZ* Ceftazidime, *C* Chloramphenicol, *IMI* Imipenem, *AK* Amikacin, *K* Kanamycin, *S* Streptomycin *GM* Gentamycin, *CIP* Ciprofloxacin, *NOR* Norfloxacin, *ERN* Enrofloxacin, *TS* Sulphamethoxazole/Trimethoprim

### PCR profiling of resistance genes

#### Prevalence of AMR genes

Results of genetic profiling of the observed phenotypic resistances among the isolates showed predominance of *bla*_ampC_ 90 %, *bla*_CMY_ 70 %, *bla*_CTX-M_ 65 %, and *bla*_TEM_ 27 %, among the isolates that were resistance to ampicillin, amoxicillin/clavulanate, cephalothin, cefuroxime, ceftazidime. PCR amplification of the *bla*_SHV_*, bla*_VEB_*, bla*_CTX*-*Mgroup1_ and *bla*_CTX*-Mgroup9*_ did not yield any amplicon. The *tet*(A) and *str*A resistance genes were amplified from 70 % and 80 % respectively of the isolates that were phenotypically resistant to tetracycline, oxytetracycline and streptomycin.

**Fig. 2:**
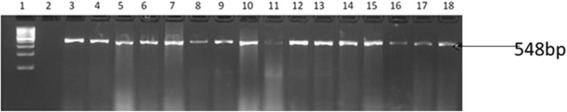
Agarose gel images of amplicons obtained from PCR with primers designed for *str*A resistance gene of *E.* *coli* isolates recovered from this study. Lane 1 is molecular size markers (100 bp), lane 2 is negative control (PCR mix without DNA) while lanes 3 to 18 are *str*A (548 bp) gene from O157 strains isolated in this study

**Fig. 3:**
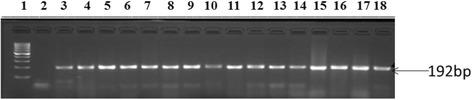
Agarose gel image of amplicons obtained from PCR with primers designed for *bla*-_ampC_ resistance gene of *E*. *coli* isolates recovered from this study. Lane 1 is molecular size markers (100 bp), lane 2 is negative control (PCR mix without DNA) while lanes 3 to 18 are bla-_ampC_ (198 bp) gene from O157 strains isolated in this study

**Fig. 4:**
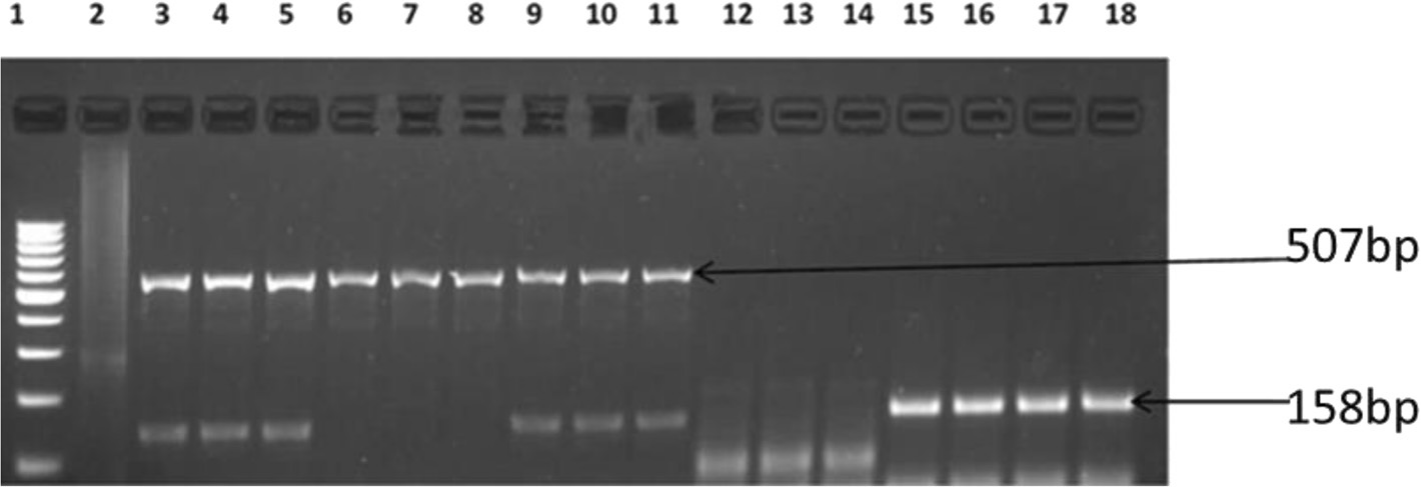
Gel image of amplicons obtained from multiplex PCR with primers designed for *bla*-_CMY_ and *bla*-_CXT-M_ resistance genes of E coli isolates recovered from this study. Lane 1 is molecular size markers (100 bp), lane 2 is negative control (PCR mix without DNA) while lanes 3 to 18 are bla-_CMY_ (507bp) and bla-_CXT-M_ (158 bp) genes from O157 strains isolated in this study

Altogether, AMR genes were detected in all *E. coli* isolates. The most frequent resistance genes were *tet(*A), *str(*A), *bla-*_ampC,_*bla-*_CMY-I_ (Table 5). With a very few exceptions, susceptibility test results were consistent with genotyping results. Gel photographs of some of the amplified resistance genes are shown in Figs. 2, 3 and 4. Overall, AMR genes were identified in 92 % of the O157 isolates recovered from studied samples. Resistance genes detected from isolates as shown in Table 5.

**Table 5:** PCR targeted genes and their percentage occurrence among the isolates

Resistance gene profiled	Percentage of amplified genes
*bla*-_ampC_	90 %
*bla*-_CMY_	70 %
*bla*_CTXM_	65 %
*Tet(A)*	70 %
*str*A	80 %
*bla*_TEM_	27 %
*bla*_SHV_	0 %
*bla*_VEB_	0 %
*bla*_CTXMgroup1_	0 %
*bla*_CTXM-group9_	0 %

## Discussions

The prevalence of STEC O157 serogroup in fecal samples collected from commercial dairy cattle was investigated. *Escherichia coli* O157:H7 (O157) is the Shiga toxin-producing *E. coli* (STEC) serotype most frequently isolated and most often associated with hemolytic uremic syndrome (HUS) in the United States [2]. According to the U.S. Centers for Disease Control and Prevention, an estimated 265,000 STEC infections occur each year in the United States. STEC O157 causes about 36 % of these infections and non-O157 STEC cause the rest [2]. STEC inhabits in the guts of ruminant animals, including cattle, goats, sheep, deer, and elk [2]. The major source for human illnesses is cattle and around 5–10 % of those who are diagnosed with STEC infection develop a potentially life-threatening complication known as hemolytic uremic syndrome (HUS) with young children and the elderly more likely to develop severe illness and hemolytic uremic syndrome (HUS) than others.

In this study, a total of 400 fecal samples were collected from two commercial dairy farms in the Eastern Cape Province of South Africa. These samples were analyzed for the presence of O157 *E.coli* strain. A total of 95 isolates confirmed by PCR targeting the O-unit flippase gene (*wzx*) were delineated to be O157 isolates. Results of the determination for the presence of Shiga toxin encoding gene (*stx*1 and *stx*2) among the 95 isolates showed that 35 (36.84 %) harbored the *stx*1 gene, 26 (27.4 %) were positive for *stx2* while 9 (9.5 %) harbored both *stx*1 and *stx*2 genes. Twenty five (26.3 %) of the isolates were commensals as no Shiga toxin genes were detected in them.

According to Gyles [3], ruminants especially cattle and sheep are the major reservoir of STEC and individual animal could carry more than one serogroups of STEC. During processing, meat derived from infected animals may become contaminated by STEC contained fecal materials if they are mistakenly mixed with it. Barlow and Mellor [27] had reported the presence of STEC in fecal samples of cattle from Australia where a prevalence of 10 % was observed with *E.coli* O157 accounting for 1.7 % of all the isolates. It is possible for fresh farm produce to be contaminated with STEC where irrigation water or soil treated with farm effluent or manure is used in growing them. A prevalence as high as 33.5 % of STEC in bulk milk has been reported internationally [28]. It is also possible for STEC to survive for a long time in soil applied with manure from cattle and sheep. The possibility of water sources being contaminated by STEC is also very high as fecal materials of animal origin could be washed through storm drains into fresh waterbodies thus posing health challenges to the people who depend on such waterbodies for several uses. WHO [29] had reported waterborne transmission of STEC in both drinking and recreational water indicating the animal fecal matters are capable of transmitting STEC producing *E.coli*.

There are increasing concerns about the use of antimicrobial products in food-producing animals and focus has been on human food safety because foods of animal origin are sometimes identified as the vehicles of food borne disease as well as resistant food borne pathogens carrying resistant genetic materials. We profiled the antimicrobial susceptibility of *E.coli* O157 isolates recovered from commercial dairy cattle that are constantly receiving (tylosin, advocin, ampicillin, tetracycline) antimicrobial agents. We observed a very high level of multiple antimicrobial resistances among the isolates and the most common resistance was to tetracycline. This is not surprising since tetracycline is often used as a first-line antimicrobial in disease prevention and growth promotion in food animals and its widespread use has likely contributed to high rates of resistance [30]. The frequency of tetracycline resistance among the *E. coli* isolates from the farms that we investigated was 96.8 %, which is within the range of values described in previous reports (68 to 93 %) [31, 32]. Genetic profiling of the resistance determinants showed that the resistances were encoded by *bla*_ampC_*, bla*_CMY_, *bla*_CTXM,_*bla*_TEM_ genes for the ESLBs (extended spectrum β-lactamases), while *tet*A and *str*A genes were responsible for tetracycline and streptomycin respectively. High prevalence of CTX-M β-lactamase–encoding genes in *Enterobacteriaceae* has been reported in stool specimens from healthy asymptomatic volunteers in a rural community in Thailand [11]. The fact that bacteria which infect animals could also establish infections in humans poses concerns about the potential spread of the *bla*-_CTX-M_ genes from food animal products to humans through the food chain. CTX-M β-lactamase has been increasingly reported in *E. coli* from various food-producing animals worldwide raising a potential threat to public health with the earliest account of CTX-M β-lactamase of food animal origin from Spain, where a CTX-M-14–producing *E. coli* was isolated from healthy chickens [33]. Since then, *E.coli* harboring CTX-M β-lactamase encoding gene has been reported from healthy cattle from Japan [34] and Hong Kong [35], and from sick or healthy cattle from France [36, 37] and in the United States [38, 39]. Similarly, *E.coli* strains isolated from pigs that have the genetic repertoire to produce CTX-M-β-lactamase have also been reported from Hong Kong [40], China [41], Spain [42], and France [36]. In this study, genetic resistance determinants were however not amplified from some of the isolates and this could be attributed to the fact that the genetic elements targeted in our PCR profiling were not responsible for the observed phenotypic resistances as there are numerous arrays of genes that encodes for resistances to the drugs. Besides, there are many resistance mechanisms like the efflux pump, intrinsic resistance, innate resistant or acquire resistance to one or few classes of antimicrobial agents. Our findings also showed that most of the isolates were susceptible to imipenem, amikacin, kanamycin, the quinolones (norfloxacin, ciprofloxacin and enrofloxxacin) and gentamicin. This finding is curious as regards the susceptibility to the quinolones because the farms that were sampled uses advocin (danofloxacin) which is a synthetic fluoroquinolone in the treatment of respiratory disease in chickens, cattle and pigs and ought to have selected for other quinolone resistances as they have similar structure and mode of action.

To understand the bases of high tetracycline resistance among our isolates, we screened for *tet* determinants. The predominance of the *tet*(A) efflux gene observed in our study is similar to that previously documented in coliforms (73 %) of human and animal origins by Marshall [43]. In this study, we observed also a very high resistance to streptomycin among the isolates which is in partial agreement with the reported 53 % resistance of *E.coli* O157 isolates from feedlots by Rao [44]. Also, Sawant [45] reported a prevalence of 93 % of tetracycline resistance in *E.coli* of dairy cattle with *tet*(B) accounting for the resistance determinant in 93 % of their isolates and this is similar to our finding though mediated by different variants of the gene.

The fact that bacteria from animals spread to the food products during slaughter and processing has been extensively documented [46–49]. The detection of *E.coli* resistant to antibiotic growth promoters (AGPs) in food products derived from animals where AGPs have been used therefore comes as no surprise. Resistant bacteria and active antibiotics, or active metabolites of antibiotics can also spread on farmland with manure. In this study, we isolated toxigenic O157 *E.coli* strains carrying _*S*_*tx*1 and *stx*2 genes that were also multi-resistant to many antibiotics some of which are medically important in human medicine. The possibility of these antibiotic resistant strains being shed into the environment and the eventual transmission of the resistance determinants to nonpathogenic environmental bacteria is high. Such transfer of resistance determinants could fuel the spread of antibiotic resistant bacteria (ARB) that could have grave implications on the health of humans and animals thus increasing the burden of disease in the community. There is therefore need for urgent policy formulations on the prudent use of antimicrobials in both human and veterinary medicine as failure in this regards could spell doom in the nearest future.

## Conclusions

In conclusion, this study demonstrated that antimicrobial resistant (AMR) determinants were present in dairy cattle exposed to veterinary antimicrobials for both therapeutic and as growth promoting agents. The overall resistance rate was high and the isolates have the genetic repertoires to survive antimicrobial pressure. Though genetic capacity for Shiga toxins production have been reported among *E.coli* nonO157 serogroups, we did not profile for any other serogroups apart from O157 in the fecal samples analyzed. The observed phenotypic multiple resistances among the isolates were also genetically confirmed. There were high β-lactam resistances among the isolates and this poses health implications as these are the drugs of choice for the management of numerous Gram negative infections. The results of this study suggest that agricultural activities, specifically antimicrobial use may have a significant impact on AMR evolution in general. More studies with larger sample sizes and more precise AMR genes typing by DNA sequencing and molecular typing of bacterial strains are needed to further throw more light in this regard.

## Abbreviations

STEC: Shiga toxin-producing *E. coli*
HUS: hemolytic uremic syndrome
stx1: Shiga toxins 1
stx2: Shiga toxins 2
TSB: trypticase soya broth
SMAC: sorbitol MacConkey agar
PCR: polymerase chain reaction
AMR: antimicrobial resistant
AGPs: antibiotic growth promoters
ARB: antibiotic resistant bacteria
